# Supplementary material for: Resolving the full spectrum of human genome variation using Linked-Reads
Source: Genome Res. 2019 Apr;29(4):635–45. doi: 10.1101/gr.234443.118 (PMC6442396; doi:10.1101/gr.234443.118)
Supplement: Supplemental Material [file supp_gr.234443.118_Supplemental_longranger-2.2.zip › longranger-2.2.tar.bz2!/lr2.2-public/lib/stan/cmdstan/stan/doc/api/html/agrad_2fwd_2functions_2falling__factorial_8hpp_source.html]

Stan: src/stan/agrad/fwd/functions/falling\_factorial.hpp Source File


|  |  |
| --- | --- |
|  | Stan  2.5.0  probability, sampling & optimization |


- Main Page
- Related Pages
- Namespaces
- Classes
- Files

- File List
- File Members

All Classes Namespaces Files Functions Variables Typedefs Enumerations Enumerator Friends Macros Pages

- src
- stan
- agrad
- fwd
- functions

falling\_factorial.hpp

Go to the documentation of this file.

1 #ifndef STAN\_\_AGRAD\_\_FWD\_\_FUNCTIONS\_\_FALLING\_FACTORIAL\_HPP

2 #define STAN\_\_AGRAD\_\_FWD\_\_FUNCTIONS\_\_FALLING\_FACTORIAL\_HPP

3

4 #include <stan/agrad/fwd/fvar.hpp>

5 #include <stan/meta/traits.hpp>

6 #include <stan/math/functions/falling\_factorial.hpp>

7 #include <boost/math/special\_functions/digamma.hpp>

8

9 namespace stan {

10

11  namespace agrad {

12

13  template<typename T>

14  inline fvar<T>

15  falling\_factorial(const fvar<T>& x, const fvar<T>& n) {

16  using stan::math::falling\_factorial;

17  using boost::math::digamma;

18

19  T falling\_fact(falling\_factorial(x.val\_,n.val\_));

20  return fvar<T>(falling\_fact, falling\_fact \* digamma(x.val\_ + 1) \* x.d\_

21  - falling\_fact \* digamma(n.val\_ + 1) \* n.d\_);

22  }

23

24  template<typename T>

25  inline fvar<T>

26  falling\_factorial(const fvar<T>& x, const double n) {

27  using stan::math::falling\_factorial;

28  using boost::math::digamma;

29

30  T falling\_fact(falling\_factorial(x.val\_,n));

31  return fvar<T>(falling\_fact,

32  falling\_fact \* digamma(x.val\_ + 1) \* x.d\_);

33  }

34

35  template<typename T>

36  inline fvar<T>

37  falling\_factorial(const double x, const fvar<T>& n) {

38  using stan::math::falling\_factorial;

39  using boost::math::digamma;

40

41  T falling\_fact(falling\_factorial(x, n.val\_));

42  return fvar<T>(falling\_fact,

43  -falling\_fact \* digamma(n.val\_ + 1) \* n.d\_);

44  }

45  }

46 }

47 #endif

falling\_factorial.hpp

stan::agrad::fvar

**Definition:** fvar.hpp:13

traits.hpp

fvar.hpp

stan::agrad::falling\_factorial

fvar< T > falling\_factorial(const fvar< T > &x, const fvar< T > &n)

**Definition:** falling\_factorial.hpp:15

stan::agrad::digamma

fvar< T > digamma(const fvar< T > &x)

**Definition:** digamma.hpp:16

stan::agrad::fvar::d\_

T d\_

**Definition:** fvar.hpp:16

stan::math::falling\_factorial

boost::math::tools::promote\_args< T1, T2 >::type falling\_factorial(const T1 x, const T2 n)

**Definition:** falling\_factorial.hpp:53

stan::agrad::fvar::val\_

T val\_

**Definition:** fvar.hpp:15


---

[ Stan Home Page ]

*© 2011–2014,
Stan Development Team.*
